# Predictors of pediatric readmissions among patients with neurological conditions

**DOI:** 10.1186/s12883-020-02028-0

**Published:** 2021-01-05

**Authors:** Ryan O’Connell, William Feaster, Vera Wang, Sharief Taraman, Louis Ehwerhemuepha

**Affiliations:** 1 Sharon Disney Lund Medical Intelligence and Innovation Institute (MI3) at CHOC, Orange, California USA; 2 Children’s Hospital of Orange County (CHOC), Orange, California USA; 3 Department of Emergency Medicine, University of California - Irvine, Irvine, California USA; 4 Neuroscience, Hamilton College, Clinton, New York USA; 5 Department of Pediatrics, University of California - Irvine, Irvine, California USA; 6 Chapman University School of Computational and Data Science, Orange, California USA

**Keywords:** Pediatric, Neurology, Readmission, Predictors, Indicators

## Abstract

**Background:**

Unplanned readmission is one of many measures of the quality of care of pediatric patients with neurological conditions. In this multicenter study, we searched for novel risk factors of readmission of patients with neurological conditions.

**Methods:**

We retrieved hospitalization data of patients less than 18 years with one or more neurological conditions. This resulted in a total of 105,834 encounters from 18 hospitals. We included data on patient demographics, prior healthcare resource utilization, neurological conditions, number of other conditions/diagnoses, number of medications, and number of surgical procedures performed. We developed a random intercept logistic regression model using stepwise minimization of Akaike Information Criteria for variable selection.

**Results:**

The most important neurological conditions associated with unplanned pediatric readmissions include hydrocephalus, inflammatory diseases of the central nervous system, sleep disorders, disease of myoneural junction and muscle, other central nervous system disorder, other spinal cord conditions (such as vascular myelopathies, and cord compression), and nerve, nerve root and plexus disorders. Current and prior healthcare resource utilization variables, number of medications, other diagnoses, and certain inpatient surgical procedures were associated with changes in odds of readmission. The area under the receiver operator characteristic curve (AUROC) on the independent test set is 0.733 (0.722, 0.743).

**Conclusions:**

Pediatric patients with certain neurological conditions are more likely to be readmitted than others. However, current and prior healthcare resource utilization remain some of the strongest indicators of readmission within this population as in the general pediatric population.

**Supplementary Information:**

The online version contains supplementary material available at 10.1186/s12883-020-02028-0.

## Background

In pediatrics, the majority of health care costs can be attributed to a small group of patients. This group of patients is typically composed of children with medical complexities who require intensive care and are not easily catered to by existing models of health [1]. Improvements in the quality of care of these patients is paramount to ensuring higher quality of life and reduced morbidity and mortality. Patients with neurological conditions largely contribute to this category as they often require complex medical care, making unplanned readmissions undesirable [2–4]. There is a dearth of research in risk factors and predictive models of unplanned readmission among these patients, so further investigation is a key step to improving quality of care and reducing corresponding readmission rates [5]. Reductions in unplanned readmissions may reduce financial distress, disruption in the families’ and patients’ lives, and help hospitals secure funding from organizations such as the Center for Medicare and Medicaid Services, which uses unplanned readmission rates as a proxy measure for hospital quality of care [6, 7].

Previous studies on unplanned readmission among patients with neurological conditions have indicated multiple anti-epilepsy drugs, pediatric intensive care unit admission, seizure with major complication or comorbidity, and presence of a major complication or comorbidity irrespective of diagnosis as factors associated with higher risk of unplanned readmission [3, 8]. In this study, we aimed to identify novel factors that may predict a higher risk of unplanned readmission in pediatric neurology and provide a prediction model that may be useful to clinicians in their efforts to reduce readmission rates [9, 10]. Previous studies were limited by sample size and only included a single center. Using Cerner Health Facts Database, we were able to access data from more than 600 facilities across all care settings from participating Cerner Corporation clients in the United States. Results from this study may add to overall knowledge of unplanned readmissions in pediatric neurology, allowing for more targeted and higher quality of care.

## Methods

We retrieved inpatient hospitalization data between 2000 and 2017 from the Cerner Health Facts Database of pediatric patients (< 18 years) with a nervous system condition as identified by diagnosis codes. The hospitals entered the study at different times and provided data over an average period of 9.2 years. We tagged each encounter as planned or unplanned using the diagnosis codes indicative of an encounter that is likely to be an aftercare or planned hospitalization. These codes included encounters for examination, encounters related to reproduction, encounters for specific health care needs such as chemotherapy, radiotherapy, surgery, and postprocedural aftercare, and encounters relating to certain mental health services, counselling, and care provider dependency corresponding to International Classification of Diseases Version 10 (ICD-10-CM) codes of Z00-Z13, Z30-Z39, Z40-Z53, and Z69-Z76 respectively. Earlier encounters used ICD-9-CM codes but were first converted to ICD-10-CM prior to our analyses of the data. We included only hospitals for which there was data in all of the database tables used for the study and for which there were at least 1000 encounters for a nervous system condition. We estimated the average age of all patients seen at each hospital and determined a free-standing pediatric hospital as one where the average age is less than 18.

We estimated readmissions within 30 days and excluded encounters which preceded a planned readmission to the hospital within 30 days. This was to ensure that we were only comparing unplanned readmissions to encounters for which there was no readmission within 30 days. We chose the 30-day metric over 7 days because it may provide increased opportunities to identify patients who potentially require interventions to improve quality of care and would help reduce the challenge in model performance due to class imbalance. We selected predictors encompassing the type/source of encounter, demographics, proxies for social determinants of health, sub-classes of neurological conditions, count of the number of other systems of diagnoses of each encounter, several measures of resource utilization (including histories of hospitalizations, readmissions, and emergency room/department visits), number of surgical procedures carried out during the visit, and the number of medications administered to the patient during the index visit. See Table 1 and the Additional file 1 for a complete list of variables considered during model development.

**Table 1:** Demographics, resource utilization, and comorbid conditions

Variables	Levels	Not readmitted	Readmitted	chi-squared or t-test *p* value
		n (%) or mean (sd)	n (%) or mean (sd)	
Age	-	6.75 (6.09)	7.08 (5.97)	<0.001
Sex	Female	71455 (46.02)	8204 (46.43)	0.301
	Male	83808 (53.98)	9464 (53.57)	
Race/ethnicity	Caucasian	88573 (57.05)	9582 (54.23)	<0.001
	African American/ Black	30498 (19.64)	3415 (19.33)	
	Hispanic	4185 (2.70)	586 (3.32)	
	Native American	2119 (1.36)	199 (1.13)	
	Asian/ Pacific Islander	2060 (1.33)	235 (1.33)	
	Other	27828 (17.92)	3651 (20.66)	
Payer	Commercial	47555 (30.63)	5242 (29.67)	<0.001
	Governmental	67552 (43.51)	8836 (50.01)	
	Self pay	2790 (1.80)	188 (1.06)	
	Others	37366 (24.07)	3402 (19.26)	
Length of stay (days)	< 2	37491 (24.15)	2708 (15.33)	<0.001
	2 - 3	55240 (35.58)	5245 (29.69)	
	4 - 6	27874 (17.95)	3812 (21.58)	
	7 or more	34658 (22.32)	5903 (33.41)	
Index visit is planned	No	123208 (79.35)	14383 (81.41)	<0.001
	Yes	32055 (20.65)	3285 (18.59)	
Admitted through ED	No	33912 (21.84)	4008 (22.69)	0.011
	Yes	121351 (78.16)	13660 (77.31)	
Index visit is a readmission?	No	139316 (89.73)	11881 (67.25)	<0.001
	Yes, unplanned	13042 (8.40)	4513 (25.54)	
	Yes, planned	2905 (1.87)	1274 (7.21)	
Previous ED visits (prior 6mo)	0	118780 (76.50)	11369 (64.35)	<0.001
	1	22665 (14.60)	3239 (18.33)	
	2	7696 (4.96)	1444 (8.17)	
	3 or more	6122 (3.94)	1616 (9.15)	
Previous hospitalizations (prior 6mo)	0	119481 (76.95)	7959 (45.05)	<0.001
	1	22409 (14.43)	3673 (20.79)	
	2	7097 (4.57)	2076 (11.75)	
	3 or more	6276 (4.04)	3960 (22.41)	
Previous readmissions (prior 6mo)	0	146664 (94.46)	13043 (73.82)	<0.001
	1	5441 (3.50)	2067 (11.70)	
	2	1727 (1.11)	1076 (6.09)	
	3 or more	1431 (0.92)	1482 (8.39)	
Number of comorbid diagnoses (by ICD-10-CM chapters)	-	5.35 (5.22)	6.73 (6.12)	<0.001

### Statistical considerations

We excluded rare neurological conditions (toxic encephalopathy (G92), transient cerebral ischemic attacks and related syndromes (G45), vascular syndromes of brain in cerebrovascular diseases (G46), and other diseases of the nervous system without clear designation (G98-G99)) as well as rare surgical procedures (endocrine, eye/ocular adnexa, and mediastinum and diaphragm surgeries) as they present problems with model development due to statistical separability [11]. We assessed multicollinearity between predictors using the generalized variance inflation factor which estimates the degree to which variance estimates are inflated due to correlation between predictors [12].

We split the data into a training (80%) and test set and developed mixed effects (random intercept) logistic regression model. We performed variable selection using a stepwise procedure to obtain the combination of predictors that result in the lowest value of the Akaike Information Criteria [13]. We assessed model performance by estimating the area under the operator characteristic curve, and values of sensitivity, positive predictive value, relative risk, and the number needed to evaluate at an a priori value of specificity at 0.90. All analyses were carried out using HealtheDataLab and the R Statistical Programming Language [14, 15].

## Results

The inclusion/exclusion criteria as discussed in the Methods resulted in 105,834 total index admissions from 18 hospitals that were evaluated in the analysis. This includes 12,737 30-day readmissions and corresponds to a 12.0% 30-day readmission rate. The mean age of the patients was 8 years with a standard deviation of 6 years. There were 52.6% males, 56.4% Caucasians, 20.4% African American/Black, and 2.6% patients of Hispanic ethnicity. The largest groups of patients were on Medicare/Medicaid health insurance (42.9%) while 31.7% had Commercial insurance, with the remainder on other health insurance types or self-pay.

We assessed univariable (unadjusted) statistics and developed a corresponding multivariable model on the training dataset. All variables considered had univariable significance (unadjusted *p* value < 0.05) except sex, demyelinating diseases of the CNS (G35-G37), epilepsy (G40), intraoperative and postprocedural complications of the CNS (G97), migraine (G43), other degenerative diseases of the nervous system (G30-G32), other headache syndromes (G43), other and unspecified diseases of the spinal cord (G95), pain (G89), systemic atrophies primarily affecting the CNS (G10-G14), surgical procedures on the digestive system, integumentary procedures, and surgical procedures on the urinary and reproductive organs. We did not detect any severe problems with multicollinearity between predictors based on a rule of thumb threshold for excluding highly correlated variables [12].

Of neurological factors associated with a change in the odds of readmission within 30-days, the multivariate model indicates that the following are risk factors of readmission among patients with neurological conditions: malignant neoplasm of the brain (C71), polyneuropathies (G62), congenital hydrocephalus (G91), other amino acid metabolism disorders (E72), paralytic strabismus (H49), hydrocephalus (G91), other autosomal trisomies (Q92), neuromuscular dysfunction of bladder (N31), microcephaly (Q02), Down Syndrome (Q90), brain disorders (G93). On the other hand, there are reduced odds of readmission among patients with some neurological conditions (Table 2) including sleep disorders (G47), convulsions (R56), and intracranial injury (S06). The remaining neurological conditions identified did not have statistically significant effects on the risk/odds of readmission in the multivariable model. The number of comorbid diagnoses (such as respiratory and circulatory systems of diagnoses) is associated with increased odds of readmission. In particular, patients undergoing chemotherapy during the index visit have highly elevated odds of readmission – these patients are being treated for some form of cancer as well as some neurological condition (that may or may not be related with the diagnosis for cancer).

**Table 2:** Multivariable Results

Variable	Levels	Odds ratio	*p* values
Index Visit is Planned	Yes	0.898 (0.847, 0.952)	<0.001
Length of Stay (days)	<2	Reference	-
	2 - 4	1.228 (1.167, 1.293)	<0.001
	4 - 6	1.441 (1.361, 1.525)	<0.001
	7 or more	1.585 (1.492, 1.683)	<0.001
Age	-	0.993 (0.99, 0.997)	<0.001
Race	Caucasian	Reference	-
	African American/Black	0.992 (0.946, 1.04)	0.729
	Hispanic/Latino	1.126 (1.016, 1.247)	0.024
	Native American	0.855 (0.696, 1.05)	0.136
	Asian/Pacific Islander	1.1 (0.948, 1.276)	0.208
	Other	0.978 (0.928, 1.031)	0.417
Sex	Female	Reference	-
	Male	1.017 (0.983, 1.052)	0.336
Payer	Commercial	Reference	
	Governmental	1.028 (0.986, 1.073)	0.196
	Self-Pay	0.805 (0.688, 0.943)	0.007
	Others	1.031 (0.972, 1.092)	0.309
Previous ED Visits (prior 6 mo)	0	Reference	-
	1	1.106 (1.056, 1.159)	<0.001
	2	1.200 (1.123, 1.283)	<0.001
	3 or more	1.297 (1.209, 1.391)	<0.001
Index Visit is a Readmission?	No	Reference	-
	Yes, Planned	1.338 (1.269, 1.411)	<0.001
	Yes, Unplanned	1.666 (1.516, 1.832)	<0.001
Previous Hospitalizations (prior 6 mo)	0	Reference	-
	1	1.695 (1.613, 1.781)	<0.001
	2	2.348 (2.18, 2.528)	<0.001
	3 or more	3.014 (2.738, 3.317)	<0.001
Previous Readmissions (prior 6 mo)	0	Reference	-
	1	1.179 (1.09, 1.274)	<0.001
	2	1.496 (1.338, 1.673)	<0.001
	3 or more	2.051 (1.831, 2.298)	<0.001
Neurologic Risk Factors	Malignant neoplasm of brain (C71)	1.953 (1.788, 2.133)	<0.001
	Polyneuropathies (G62)	1.5 (1.248, 1.803)	<0.001
	Congential Hydrocephalus (Q03)	1.318 (1.175, 1.478)	<0.001
	Other AA Metabolism Disorders (E72)	1.313 (1.095, 1.575)	0.003
	Paralytic Strabismus (H49)	1.288 (1.124, 1.476)	<0.001
	Hydrocephalus (G91)	1.218 (1.131, 1.311)	<0.001
	Other Autosomal Trisomies (Q92)	1.214 (1.019, 1.448)	0.030
	Neuromuscular Dysfunction of Bladder (N31)	1.155 (1.034, 1.29)	0.011
	Microcephaly (Q02)	1.131 (1.005, 1.272)	0.041
	Down Syndrome (Q90)	1.129 (1.036, 1.23)	0.005
	Brain Disorders (G93)	1.078 (1.019, 1.14)	0.009
Other Neurological Conditions	Convulsions (R56)	0.922 (0.874, 0.972)	0.003
	Nervous & Msuculoskeletal System Symptoms (R29)	0.882 (0.781, 0.995)	0.041
	Coginitive Function Symptoms (R41)	0.853 (0.776, 0.938)	<0.001
	Sleep Disorders (G47)	0.84 (0.791, 0.892)	<0.001
	Meningitis, Other Causes (G03)	0.829 (0.698, 0.985)	0.033
	Nystagmus & Irregular Eye Movements (H55)	0.825 (0.686, 0.992)	0.040
	Newborn Cerebral Distrubances (P91)	0.822 (0.705, 0.958)	0.012
	Speech & Language Development Disorders (F80)	0.811 (0.725, 0.907)	<0.001
	Somnolence, Stupor, Coma (R40)	0.786 (0.694, 0.889)	<0.001
	Mental Disorders Due to Physiological Condition (F06)	0.768 (0.648, 0.91)	0.002
	Bacterial Meningitis (G00)	0.622 (0.487, 0.796)	<0.001
	Newborn Muscle Tone Disorders (P94)	0.621 (0.567, 0.68)	<0.001
	Intracranial Injury (S06)	0.476 (0.418, 0.543)	<0.001
	Viral Meningitis (A87)	0.446 (0.353, 0.563)	<0.001
Number of Medications	-	1.014 (1.012, 1.016)	<0.001
Free-standing pediatric hospital	Yes	1.586 (1.103, 2.28)	0.013
Surgical procedures	Cardiovascular	1.204 (1.109, 1.307)	<0.001
	Musculoskeletal	0.685 (0.6, 0.781)	<0.001
	Nervous System	1.119 (1.029, 1.216)	0.009
Number of Comorbid Diagnoses (by ICD-10-CM chapters)	-	1.01 (1.006, 1.014)	<0.001
Emergent Admission	Yes	1.129 (1.079, 1.18)	<0.001

Results from the multivariable model indicate that current and prior healthcare resource utilizations are most predictive of the risk of readmission after the index visit. Patients with longer lengths of stay are more likely to be readmitted than those with shorter lengths of stay, and the higher the number of previous hospitalizations (and the longer the lengths of stay of these previous hospitalizations) within the prior 6 months, the more likely a 30-day readmission will occur after the index/current hospitalization. Similarly, the risk of subsequent readmission is elevated if the index visit is a readmission within 30-days of the prior visit. The number of previous unplanned readmissions as well as emergency room/department visits within the prior 6 months are associated with increased risk of readmission, whereas the risk of readmission is reduced if the index visit is a planned encounter. These results are consistent with prior studies mostly in the general pediatric and adult populations [3, 10, 16].

In this population of pediatric patients with neurological conditions, younger patients are more likely to be readmitted than their older peers. The type of admission is associated with a statistically significant change in the risk of readmission. Emergent admissions are associated with higher risk of readmission. In comparison to patients with Commercial insurance, self-pay patients are less likely to be readmitted.

The odds of readmission are increased among these patients with underlying neurological conditions if they are also undergoing nervous or cardiovascular system surgical procedures. On the other hand, surgical procedures on the musculoskeletal system are associated with reduced odds of readmission. The odds ratios and corresponding 95% Confidence Intervals are given in Table 2.

The area under the receiver operator characteristics curve obtained in the independent test set was 0.733 (0.722, 0.743). Based on previous studies, we recommend setting the predicted probability threshold for flagging a patient at high risk of readmission (and requiring interventions) at a value that guarantees a model specificity of 0.90 (or 90%). At this value of the specificity of the model, the sensitivity, positive predictive value, relative risk, and number needed to evaluate are 0.373 (0.354, 0.392), 0.339 (0.321, 0.356), 3.878 (3.617, 4.157), and 3 respectively. This implies that we expect to capture 37% of readmissions in such a way that there will be 2 false positives for every 1 true positive prediction. The corresponding predicted probability of readmission should be set at 0.200 (nearly twice the baseline readmission rate of these patients).

## Discussion

In this study, we identified several neurological conditions that increase the risk of readmission among pediatric neurology patients. Among the neurologic risk factors we identified, it is likely that higher readmission rates in these populations in part represents the disease burden and complex nature of presentation in these patients, rather than any specific diagnostic or therapeutic shortcomings. Specifically, a preponderance of these risk factors are chronic, lifelong diagnoses, or carry long term morbidity implications (microcephaly, Down Syndrome, metabolic disorders, etc.). This thought remains true when juxtaposed to the neurological conditions we identified to have lower odds of readmission (meningitis, intracranial injury, sleep disorders, etc.). Reduced odds of readmission among these patients may attributable to the fact that they tend to be time limited and are more frequently encountered in diagnostic isolation or with fewer comorbid complications. While many of the seemingly protective conditions can be deadly, we controlled for this by excluding patients that died during an admission. This shows one of our study limitations. We had no way of measuring the objective severity of an illness such as meningitis. An assumption could be made that very severe presentations are less likely to leave the hospital until providers are exceedingly comfortable with the patient’s recovery. Similarly, more complex presentations may lead to a lower likelihood of discharging a patient prematurely, accounting for the general trends in both the protective and risk related diagnoses we identified.

Other predictors of higher risk of unplanned readmission (among patients with underlying neurological conditions) in our multivariable model include longer length of stay, higher number of previous hospitalizations, and unplanned index visit. As representative markers of health care utilization these are consistent with findings from prior studies. Certain surgical procedures also predicted higher risk of unplanned readmission and included nervous and cardiovascular system procedures during hospitalizations. We also observed a reduced risk in patients with older age, self-pay coverage, and patients undergoing surgical procedures of the musculoskeletal system. The reduced risk from musculoskeletal procedures likely represents the specific limited nature of these encounters. Overall, current and prior healthcare resource utilizations are most predictive of risk of unplanned readmission after the index visit.

Previous studies have identified risk factors such as higher severity of illness, public health insurance, multiple antiepileptic drugs, intensive care admission, and seizures with major complication/comorbidity [3, 8]. These findings are generally consistent with the results of this study. Several proxies for severity of illness and comorbidity were all associated with increased risk of readmission. While one of the studies found that multiple antiepileptic drugs are associated with increased risk of readmission [3], our findings indicated that the higher the overall number of medications administered the higher the risk of readmission.

The aim of such research is to develop a model that may be helpful in improving our understanding of risk factors of readmission in pediatric neurology, and for predicting future risk of readmission in a clinical setting. We provided information on how this model may be implemented to assist decision making and allow for earlier interventions. Reducing unplanned readmission rates would benefit all parties involved by saving time and money and helping the patient avoid unexpected time in the hospital. Those who are determined to be at high risk according to this model may benefit from personalized interventions before and after discharge.

While the primary goal of implementing a predictive model such as this is to prevent readmission by providing additional information during an ongoing hospital stay, the results are also likely to be informative to ambulatory care providers post-discharge. A patient being flagged as high risk for readmission could help guide decision making at early post-discharge follow-up appointments, provided they are sharing information systems or interfaces with the admitting facility. Furthermore, the results should generalize to non-US healthcare settings with electronic medical records as the risk factors are not peculiar to US population.

A limitation of this study is that we could not track patients across hospitals. In other words, patients readmitted to a hospital different from the index encounter were likely not captured. This study limitation may also account for the finding that free standing pediatric hospitals are correlated with an increased risk of readmission. There are likely cases in which a pediatric patient was initially admitted at a general/adult facility are subsequently transferred to, and followed by, a dedicated pediatric facility for a higher level of care. This would be associated with a concentration of complex cases being encountered at dedicated pediatric facilities compared to non-pediatric facilities.

An additional minor limitation is the fact that the data consisted of health care systems using the Cerner EMR and who consented to sharing deidentified data. There is concern that an inability to reliably track death subsequent to discharge in our data set may impart some protective bias for some highly lethal disease states, however we have not clearly identified a suggestion of this bias in the model.

The large size of the study and distribution across the United States indicate that the findings may be helpful to a large number of pediatric neurology patients across the country. Furthermore, the opportunity of capturing 37% of unplanned readmissions with only 2 false positive for every true positive prediction is quite impressive. In other words, a large number of unplanned readmissions may be captured with high accuracy, allowing for focused interventions specifically on patients most in need of such interventions. The model specificity may be traded for higher sensitivity at hospitals where there are sufficient clinical resources for interventions. While a high false positive rate is present in our model, we focused specifically on readmissions, and further research is necessary to find if these false positive cases may be predictive of some other state that warrants additional consideration or intervention.

## Conclusions

Pediatric patients with certain neurological conditions are more likely to be readmitted than others. However, current and prior healthcare resource utilization remain some of the strongest indicators of readmission within this population as in the general pediatric population. After accounting for prior healthcare utilization, pediatric patients with malignant neoplasm of the brain, congenital hydrocephalus, microcephaly, down syndrome and other brain disorders among others have a higher risk of unplanned readmission. Our findings will allow clinicians to identify patients most vulnerable to future unplanned readmissions and adjust their care accordingly.

## Supplementary Information

**Additional file 1.** Summary statistics of neurological conditions considered.

## Data Availability

Database used for this study is closed to public access.

## Abbreviations

AUROC: Area under the receiver operator characteristic curve
NNE: Number needed to evaluate
CPT: Current Procedural Terminology
ICD-10-CM: International Classification of Diseases, Tenth Revision, Clinical Modification
